# If You're Not Thinking About Intellectual Property, You’re Not Thinking About Impact

**DOI:** 10.1049/enb2.70001

**Published:** 2025-11-07

**Authors:** Sara L. Holland

**Affiliations:** ^1^ Potter Clarkson LLP Nottingham UK

**Keywords:** innovation, scale‐up strategies, synthetic biology

## Abstract

Many of us went into research to do something good—to cure cancer or to save the world—and in engineering biology, we really are doing that, solving some of the world's most pressing problems, from plastic degradation to chemical manufacture to built‐environment solutions to next‐gen therapeutics. Or, as academics, we *think* we are doing these things. We do the research. We publish the research. Job done. But are we actually having the impact we set out to have? Or is your next big (or small) publication one of the reasons your invention never makes it to market? This short article will help you see your research in the context of real‐world innovation and give you an insight into what it takes to turn that academic work into something we can hold and use. We will look at some fundamentals of patents and what you, as scientists, should be aware of. We will then look at innovation trends in engineering biology across a range of sectors—what are the hot fields in engineering biology commercialisation and what can we use this information for. If you’re not thinking about IP, you’re not thinking about impact.

## Graphical abstract

Engineering biology needs to be commercialised to get out of the lab and into the hands of us all. The article sets out key points that scientists should know about IP, then dives into innovation trends in engineering biology through in‐depth patent analytics.

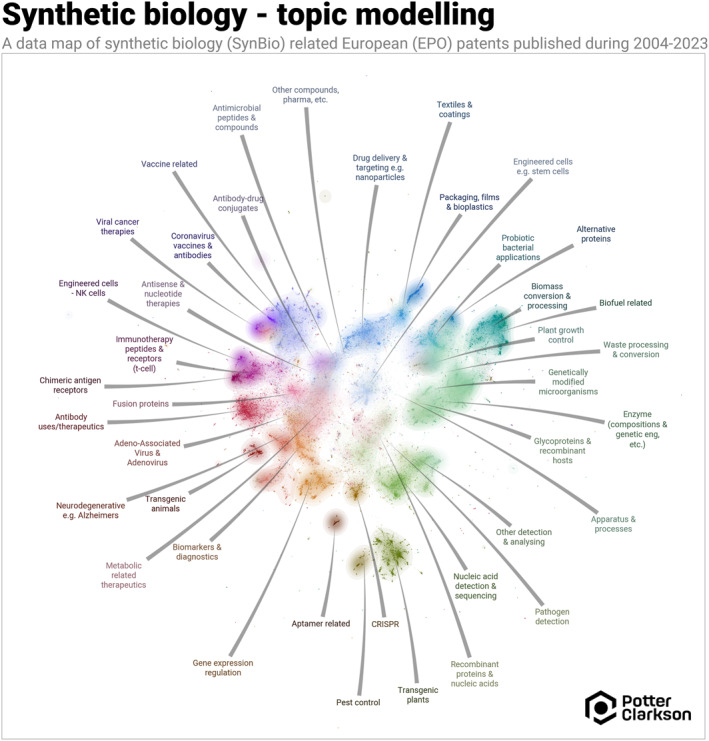

## 1 Academic Research to Real‐World Products/Services

Far too often, ideas with potential to do good in the ‘outside world’ get stranded in the academic literature. Why? Because scientists are focused on a journal's impact factor and improving their *h*‐index. Because no one thought about intellectual property (IP) before publishing. Because we think ‘someone else’ will read our research and take on the job of turning it into that climate‐change mitigating product or anti‐cancer wonder drug. And because, you know, patents are ‘bad’ aren't they? They restrict access to research, making someone a lot of money at the expense of huge populations of people—right?

The truth—the very simple truth—does actually come down to money. Like it or not, to turn your research into a marketable product that we can all use and benefit from costs money. The people running the company and the scientists and other team members all need to eat and pay bills. The consumables and equipment and lab space all cost money. The regulatory burden, for example, to get through clinical trials, costs a *lot* of money. Whether you are a big pharma company needing to turn a good profit to feed back to shareholders or a nonprofit NGO, we all need to eat and so we all need money. This is not a bad thing—it is a fact of life.

But why does needing to make money mean you need to think about IP?

Early on, you might not need to (other than really you do, because later on you will wish you had got it right from the start). There are lots of different places that early‐stage start‐ups can turn for the initial funding they need to secure lab space and the important proof‐of‐concept data. If they are lucky, the founders may have their own capital they can use and can ‘boot‐strap’ for a while. Often start‐ups apply for government funded equity free grants (e.g., in the UK grants awarded by Innovate UK) and exist for so long. None of these particularly require you to have any IP strategy in place.

But at some point, for example, when the company wants to scale‐up, begin trials and hire staff, it is highly likely that the company will need to raise larger amounts of capital from investors (of which there are many different types). Or the company gets to a point where they want to be acquired by a bigger company with the resources to continue with product development. At this stage, if the company has not already thought about IP and put a good strategy in place, perhaps even some years earlier, then the company will struggle to raise the investment or close the deal.

Why? Because the whole point of IP, including patents, is to ‘protect your space’—patents, for example, give the patent holder the ability to stop anyone else from doing what your patent covers in the relevant territory. Investors and potential acquirers will want to know that once they have spent all their cash developing your wonder drug, nobody else can copy it. They want to know that other companies cannot take a shortcut, circumvent all the hard R&D, all the money spent and cut right to the finished product. Investors need to make money too, just as a founder needs to have money to pay their teams. So, although in the very early days a company may be bootstrapping and not yet engaging with investors, it needs to think about IP from day one to put things in place to present a sensible approach to investors some time later. Particularly, in patents, it is very difficult and generally impossible to retroactively put something in place as many early mistakes are unfixable. And of course, surely, no founder wants their technology to be copied, regardless of whether there are investors involved or not.

There is still a clear disconnect between what we think we are achieving as academics by publishing in academic journals and the actual practical outputs of our research that reach the everyday consumer on the street. This is costing us both financially and in impact. If we want synthetic biology to fulfil its promise, we need to stop treating commercialisation like a dirty word.

This is changing though. There is a growing recognition from the government as well as amongst scientists of the role that the academic community plays in addressing global challenges. The recent Tenu USIT guide [1] sets out a ‘landing pad’ of terms for university knowledge exchange departments to initiate spinout agreements with the aim of streamlining often very painful and lengthy spinout negotiations; and an announcement in May 2025 that the UK government is putting £30 million into university clusters across Merseyside, East Anglia, Northeast England and the Midlands to commercialise academic research [2].

The other, perhaps less altruistic but equally valid reason to consider commercialising your research, is to give yourself a career. There is an increasing realisation amongst academics that at some point, most of them will have to go and do something else—there are simply not enough positions at the top for every PhD student or postdoc to become the PI of their own labs [3]. Turning your academic research into something we can all benefit from is an opportunity more researchers are taking.

So, what do you need to know?

## 2 Key IP Points for Scientist‐Innovators

Intellectual property, obtaining registered rights and enforcing them are all fairly complicated and it takes a patent attorney several years to qualify to act before the relevant patent offices. However, there are a few key things that every scientist and engineer should know and ought to think about *before* publishing research—if you do not, then the option to file a patent application and commercialise your research could very well be lost. Make publishing without first filing a patent an active decision, not an accidental mistake.

There are a range of different IP rights that protect different facets of a product or service:

- A *patent* protects your technical invention (how it works or the clever thing it does).
- A *trade mark* protects your brand (logos, name, colour scheme).
- A *design right* protects what something *looks* like (e.g., packaging, shape of a bottle).
- A *trade secret* protects the things you want to, and can, keep confidential (e.g., the various famous recipes of well‐known food and drinks).

Engineering biology is a very technical field and generates lots of new and clever things, and typically the most appropriate IP right is a patent (with some trade secrets inevitably forming parts of the overall IP strategy).

Patents protect the technical aspects of a product or method—what we are protecting has to do something or be able to be used in some way. In engineering biology, we can use patents to protect many different things—including promoters and other regulatory elements, engineered proteins, engineered strains, naturally occurring strains or proteins, products made from strains, new drug delivery vehicles, gene circuits, nucleic acid‐based methods, epigenetic patterns, medical uses and combinations of known entities. This is only a very short list. A granted patent means that you are able to stop other parties from using your promoter, or selling or making your engineered strain, giving *you* the commercial advantage.

**FIGURE 1:**
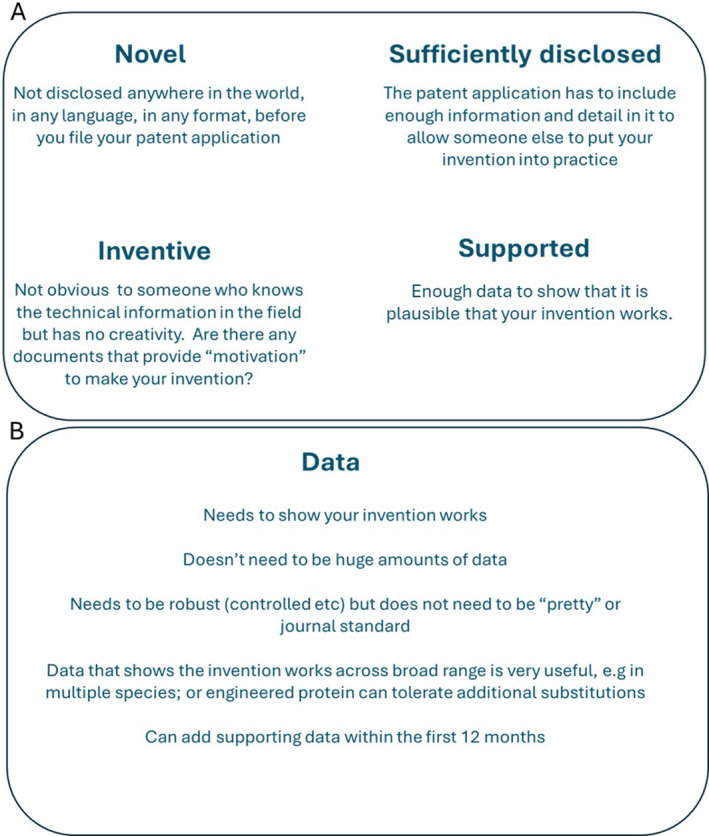
(A) Key features your invention and patent application have to demonstrate to be granted a patent; (B) information on the data requirements and standards to support a patent application.

The key things your invention needs to have to be granted a patent are set out in Figure 1—novelty, inventive step, sufficiency of disclosure and it has to be supported (i.e., some data to show it does what we are claiming it does). The key one here—particularly for academics who may publish their work—is *novelty*. For your invention to be novel, it has to be different (just one small difference is enough) from everything made public anywhere else in the world and in any language before the filing date of your patent application. Importantly, for the engineering biology community, this includes journal articles, poster presentations, oral presentations, iGEM wiki pages and theses—anything you write or say and that is made public can be harmful to a later filed patent application. As soon as your journal article is published, you can wave goodbye to the chance to file a patent application—your invention is no longer new (although some countries such as the United States have ‘grace period’ provisions—not many countries do have these provisions, and most engineering biology companies will be wanting to take a global view in terms of IP protection. Patent protection only in the United States is often not enough).

The good news is that usually once you have filed a patent application, you are free to make your invention public (though in a business context you might not want to do this for various reasons). So, it is possible to patent and publish, just always remember to file a patent application first. It is also possible to do a good and engaging scientific presentation without giving away the key aspects of your invention, keeping these confidential and protecting the novelty of your innovation.

Similarly, it is possible to both remain in academia and found/work in a biotech start‐up. Many PI's understandably do not want to relinquish their hard‐earned position at the University but also want to be involved in the exciting start‐up world. Many do this successfully and juggle the obligations to their lab, and to the company, being clear what ‘hat’ they are wearing at each moment. On the other hand, investors do like to see a committed ‘all in’ founder—a good combination is a motivated PhD student or postdoc who will run the company full time, often as CEO, with the PI acting as a part‐time advisor.

The other key aspect of IP that academics should be aware of is ownership. If you are a postdoc and you have an idea based on your research or come up with something amazing during your postdoc—who owns the IP? Not the postdoc.

The inventor is usually the first owner of an invention. An inventor is someone that came up with or developed the core inventive concept. It is not someone who just did a lot of lab work under the instruction of someone else. However, in most countries (including the UK), there is an overriding national law, which means that an employer will automatically own an employee's inventions. Because of this, most academic inventions will be owned by the host institute. This can differ between institutions and certainly can differ when it comes to undergrads, Master's and PhD students—they are not technically employees, but each will have some agreement they entered into when they registered at the university. There are some circumstances under which you as the employee/student can reasonably be said to own the invention. The advice is to always check the contracts you signed (preferably before doing so) for any IP clauses and discuss with your tech transfer office.

But it is not just about theory. If we want to understand what kind of science actually gets turned into real‐world products, we need to look at the data. Analysing patent filing data can reveal where people are putting money and pushing technology towards commercialisation.

So what does the synthetic biology patent landscape tell us about where the field is heading and how can you use that insight to shape your own IP and impact strategy?

This is not just useful for investors or government agencies. If you are a postdoc thinking about spinning out or a PI trying to map out your next grant proposal, these patent trends offer some direction, showing you where opportunity lies, which spaces are very crowded and can highlight areas that you should look into.

## 3 Real‐World Impact in Engineering Biology

Since engineering biology companies tend to file patent applications, at least for the reasons already discussed, analysing patent filings gives valuable insights into who is doing what and where. Although a review of the academic literature can show us trends in academically funded research, it cannot tell us what of that research is being commercialised. It cannot give us any insights into investor trends—what sectors using synthetic biology are being funded outside of academia? It cannot give us insights into the effects of government policy—for example, have improvements in regulatory clarity encouraged more innovation in a particular sector? Although we know that correlation does not equal causation, there are some consequences of various factors that can be picked up in patent filing trends. Patent filing trends can also be used to predict where interventions may be needed—for example, an increase in filings relating to the built environment or alternative protein might mean regulatory bodies need to be prepared for the increased approval of relevant products.

The analytics can also help those wanting to move into a particular space—is it already crowded? Are you better off looking into a less‐busy area, which may give more scope both for freedom‐to‐operate and commercial ‘uniqueness’?

**FIGURE 2:**
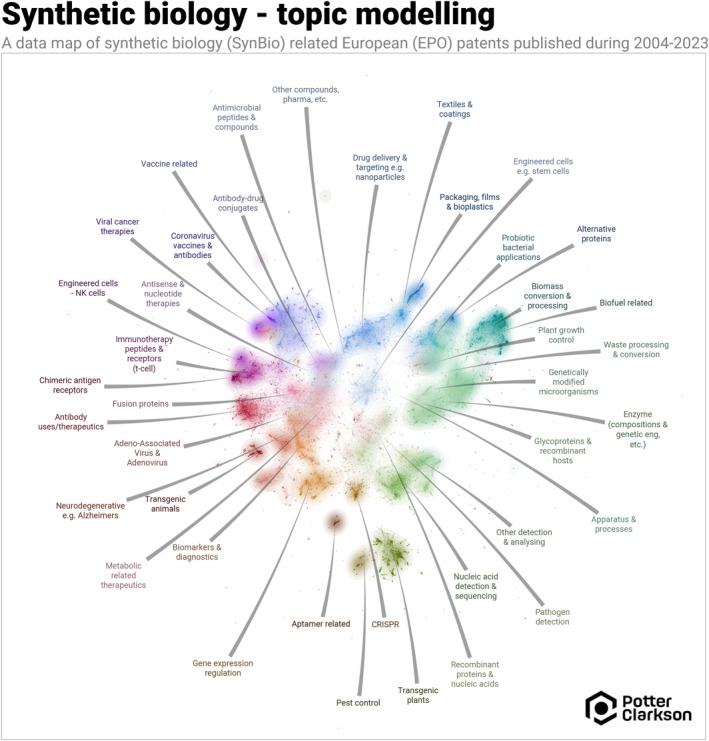
SynBio patent landscape clusters at the European Patent Office (EPO) published 2004–2023 Methodology—this study applies advanced topic modelling, optimised by hyperparameter tuning to deliver deep insights, leveraging transformer‐based text embeddings and state‐of‐the‐art natural language processing (NLP) techniques. Notably, the project used advanced machine learning techniques, including PatentSBERTa—a version of the BERT transformer model fine‐tuned on patent data—to generate embeddings (numerical representations) of ∼100,000 SynBio European patent texts. The process involved summarising key sections (title, abstract, claims) to train the model, which required significant computational time due to the contextual depth BERT provides. Using these embeddings, a bespoke topic modelling approach with BERTopic and density‐based clustering was applied, allowing for dynamic topic discovery without pre‐defining the number of clusters. Human review was crucial for labelling topics accurately, achieving ∼70%–75% accuracy in patent‐to‐topic assignments. Finally, data cleaning and expert input refined the topics (settling on 40 total), enabling more nuanced classification and trend analysis across overlapping areas like recombinant proteins and bioplastics.

Potter Clarkson, in conjunction with Inevus Advanced Analytics, has conducted a bespoke, in‐depth analysis of European patent filings in synthetic biology that published between 2004–2023 [4]. The methodology is set out in Figure 2, along with an image of the patent landscape clusters. The landscape shows a good and appropriate clustering of therapeutic‐related uses of engineering biology towards the left (antibody uses/therapeutics, immunotherapy peptides etc.) and the nontherapeutic ‘planetary health’ applications of synthetic biology (biofuels, alternative proteins, etc.) towards the right. The definition of synthetic biology used was relatively broad to encompass the whole repertoire of technology areas in which synthetic biology may be applied.

As with any patent analytics, the results come with some caveats. Clearly patent filings cannot tell us anything about innovation that is kept confidential (i.e., as a trade secret). However, unlike in some tech areas, (e.g., software, digital businesses), most companies operating in this space will file one or more patent applications at some point. It is also important to know that patents are published 18 months after they are filed—this means there is at least an 18‐month lag in the data versus what is happening now with new patent filings. The current report has a cut‐off publication date of the end of 2023. The report will be updated towards the end of 2025, adding almost 2 years of extra data.

Finally, in this case, only filings made at the European Patent Office were analysed to keep the number somewhat manageable. This means that, for example, companies filing only in China or the United States will not be captured in this dataset. However, Europe is within the top five territories for patent filings [5], and biotech companies having a serious IP strategy and plan for growth outside of the home territory will tend to seek patent protection in Europe.

## 4 Overall Trend

**FIGURE 3:**
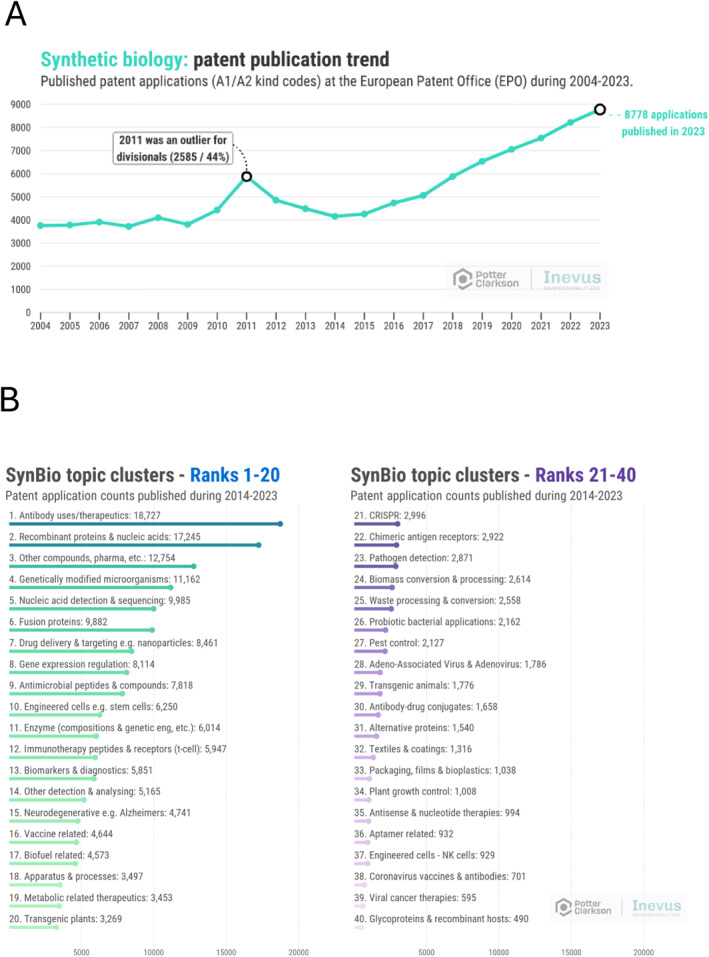
(A) The publication trend of synthetic biology‐related EPO A1/A2 filings published during 2004–2023; (B) the topic modelling carried out identified 40 diverse clusters which have been ranked based on the total number of published applications. The figure shows the ranking based on the most recent 10‐year publication period (2014–2023).

The overall trend for patent filings in engineering biology is positive, showing a steady increase in the number of European patent application publications per year since 2014, from a previously very flat base (Figure 3A). 2013 really does seem to be a pivot point for synthetic biology innovation, the patent statistics providing tangible evidence of the effect of the well‐known convergence of an increased ability to carry out targeted gene edits and molecular biology tools (e.g., CRISPR, Golden Gate assembly and Gibson Assembly); availability of very cheap DNA sequencing (just 0.05 USD per Mb DNA in 2014 vs. approximately 500 USD just 7 years earlier [6]); machine learning and AI; favourable policy changes and government funding into synthetic biology around the world (including the creation of Synthetic Biology Research Centres in the United Kingdom in 2013/2014 and inclusion in the UK's ‘Eight Great Technologies’ strategy [7]; the DARPA ‘Living Foundries’ programme launched in the Unied States and scaled in 2013 pumping money into defence and manufacturing applications of synthetic biology [8]); emergence of dedicated synthetic biology companies (Gingko Bioworks, Zymergen, Synthace); and the general recognition that biotechnology is not just for human health—it can contribute significantly to mitigating the challenges that the planet faces.

## 5 SubTopic Trends

The model breaks the overall filings down into 40 subtopics set out and ranked in Figure 3B. Since the use of engineering biology to tackle planetary health challenges is still in its relative infancy, it is unsurprising that the therapeutic uses of synthetic biology dominate the patent filing landscape with subtopics such as *Antibody related technology*, *Drug delivery*, *Antimicrobial peptides*, *Immunotherapy*, *Biomarkers and diagnostics* and *Vaccines* appearing in the top 20 topics in which applicants filed in the period 2014–2023. However, the category *Genetically modified microorganisms* does feature in Position 4, demonstrating a good level of innovation in the nontherapeutic space (although there is of course some overlap between a GMO and a therapeutic application thereof), with biofuels and transgenic plants also appearing in the top 20.

**FIGURE 4:**
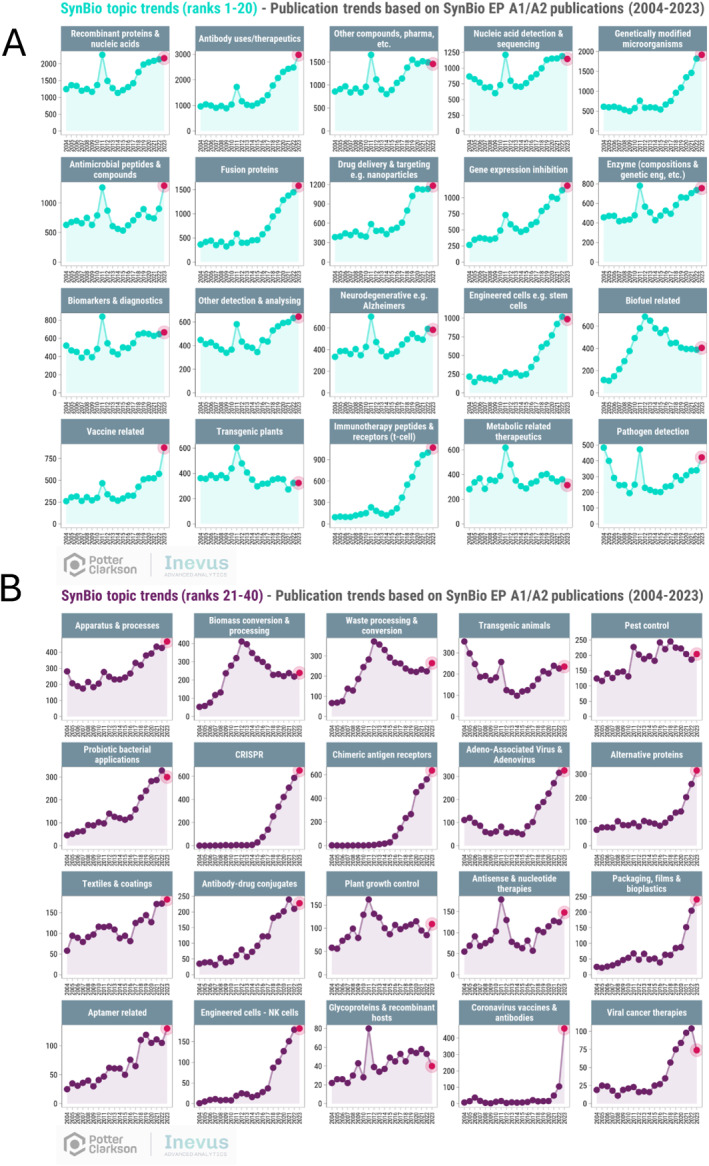
The publication year trends of SynBio technology clusters ranked (A) 1–20 and (B) 21–40, published applications during 2004–2023.

Figure 4 gives a detailed breakdown of the *per year* European patent application publication figures and reveals much more information about trends than does the overall rankings of Figure 3 (*n.b.* the peak in about 2011 was due to a short‐lived rule change at the European Patent Office which introduced a deadline for filing divisional applications and can be largely ignored). Note that the values on the *y*‐axis are different, but it is still possible to compare trends across different topics.

The *Vaccine* and *Coronavirus vaccines and* *antibodies* subtopics provide nice positive controls with patent publications soaring in 2023, reflecting patent filings made at the onset of and during the COVID‐19 pandemic (as set out above, patent applications are only published 18 months after filing); for European patent applications that have gone via the international filing system route (i.e., have filed a Patent Cooperation Treaty [PCT] application), they will not show in the European patent filing dataset until 2.5 years from the initial filing date. Of note, filings relating to GMOs show a steady and steep increase; filings relating to nucleic acid detection and sequences appear to have plateaued; and antimicrobial peptides and compounds seem to have a recent upturn in activity. Filings in drug delivery and nanoparticles also look to have plateaued—is this because the field is dominated by a few key players holding significant patents in lipid nanoparticle technology? Is there room to innovate in this space?

The subtopic ‘Glycoproteins and recombinant hosts’ is ranked 40th, with just 490 related patent filings in the last 20 years. The UK's funding body UK Research and Innovation recently committed £12.3 million to the University of Nottingham‐lead GlycoCell Engineering Biology Mission Hub (one of six newly funded hubs) [9]; therefore, it will be interesting to see if this translates to an increase in patent filings in this space over the next few years.

The full report [4] includes a detailed analysis of many of the 40 topics shown in Figure 3B. Key findings relating to *Biofuels* are discussed below.

### 5.1 Biofuel Deep‐Dive

*Biofuels* show an interesting trajectory—peaking in 2013 with a steady decline in annual filings since then. Early policy support, particularly in mainland Europe, with the deliberate aim of increasing revenue for farmers, encouraged a rapid initial uptake of biodiesel and other first‐generation biofuels, likely accounting for at least some of the sudden innovation in the biofuel space. Probably inevitably, the space became crowded, with less space for innovation, increased competition for funding and diversion of funds to maintaining the existing IP portfolio rather than filing on new innovations. Furthermore, in around 2011, policy uncertainty and unease around the future of biofuels arose following a report highlighting concerns about land use [10], with caps on food‐based (first generation) biofuels being introduced in later years, all undermining confidence in the sector. Around this time, many EU countries were also phasing out or capping subsidies for conventional (food‐based) biofuels, which may be part of the reason for the consistent decline in patent filings in Europe. The decline in patent filings in *Biofuels* is likely at least partly a direct consequence of the general concern about the use of land, and in particular, food crops, for fuels, emphasising that words do have power, and policy makers, investors and other key players that give confidence to a sector need to be mindful of the rhetoric for fear of inadvertently creating the equivalent of a bank run. To mitigate the effects of relying on food crops for biofuel generation, a large amount of research and associated patenting shifted towards so‐called ‘next‐generation’ biofuels derived from lignocellulosic biomass—the things we do not want to eat; however, uptake from this sector has been slow due to high capital expenditure requirements, challenging economics and the technical difficulties in scaling production from such recalcitrant materials to produce enough product and enough product at the right price. As with many high‐volume low‐price commodity products that we *could* make through synthetic biology, biofuels have to compete on price with heavily subsidised oil‐based alternatives, which artificially lower the cost of the incumbent products and exacerbate the difficulties in producing the synthetic biology alternatives (biofuels, chemicals, materials, etc.) at scale and at a cost that the market will take. However, as discussed below, a recent upturn in patent filings that relate to both biofuels and waste biomass conversion could indicate that the policies are doing what was intended—moving us from food crops to waste biomass as feedstock while creating a new space for innovation.

**FIGURE 5:**
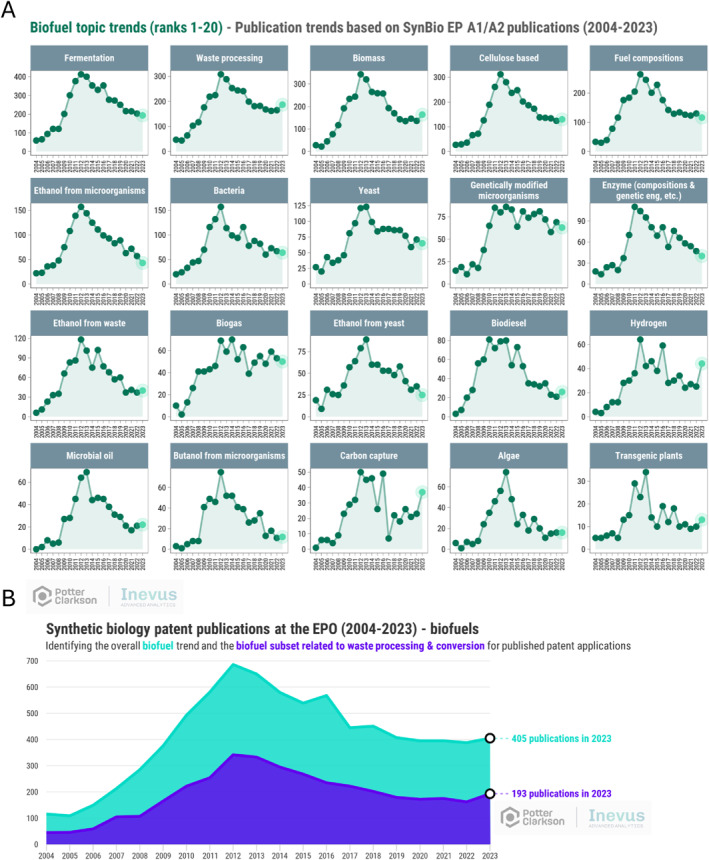
(A) Subtopics within the biofuel topic—the publication year trends of patent publications identified as relevant to biofuels and the named topic, 2004–2023. (B) Comparison of patent publications at the EPO that relate to biofuels and which relate to both biofuels (light green) and waste processing and conversion (dark blue).

From Figure 4, we can see that in recent years, the decline in *Biofuels* filings has stopped. Due to the nature of the analysis performed, it is possible to categorise a single patent publication into more than one topic. Figure 5A shows the filing trends of patent publications that are *Biofue*l related and which are also, for example, related to *Fermentation* or *Cellulose based*. Many of the trends mirror the overall *Biofuel* trend, with a historic peak around 2011 and subsequent steady decline.

Figure 5B focuses on the combination of *Biofuels* and *Waste Processing and Conversion* (green showing the total number of filings in the *Biofuels* category and blue showing those within the *Biofuels* category which also appear in the *Waste Processing and Conversion* category). Patent filings in *Biofuels* actually *increased* in 2023 relative to 2022 by 4.4%, and filings that relate to *Waste processing* and *Biofuels* increased by 19.1% in 2023 relative to 2022 (again, reflecting new patent filings likely made in 2020 or 2021), both suggesting a shift to the production of biofuels from waste biomass, in line with the policy objectives. This shift also illustrates a broader point—that external factors such as subsidy regimes, technology maturity and the economic environment for scale‐up investment all shape not only interest but the ability to sustain commercialisation of large‐scale applications.

There are some interesting recent increases that will warrant further investigation and monitoring. For example, *Biofuel* patent publications that relate to *Hydrogen* show a recent increase, suggesting that efforts to commercialise the production of hydrogen, perhaps through microbial fermentation, are an area of commercial interest. Similarly, patent publications in the area of *Biofuels* and *Carbon capture* are increasing. Both of these are perhaps not surprising, since they are areas for which comprehensive and viable solutions have not yet been realised with the chance of high reward.

In this sense, biofuels provide a worked example of a much wider phenomenon: many bio‐based chemicals, materials and energy carriers must overcome similar scale‐up and economic hurdles, although the rapid increase in the packaging, films and bioplastic subtopic may hint at opportunities for specialised products and components where niche economics are more favourable.

The filing trends of ‘Biomass conversion and processing’ and ‘Waste processing and conversion’ mirrors that of *Biofuels* (Figure 4). Historically, the major use of biomass was for conversion into ethanol for fuel. *Biomass conversion and processing*; *waste processing and conversion*; and *packing*, *films and bioplastics* feature at Positions 24, 25 and 33, respectively (Figure 3). It will be interesting to see how the position of these categories changes over time as the use of waste feedstocks for conversion to products other than fuels is being pushed by the number of bodies, particularly in the United Kingdom by the Bio‐based and Biodegradable Industries Association (BBIA) [11] and the recently announced Carbon‐Loop Sustainable Biomanufacturing Hub [12].

**FIGURE 6:**
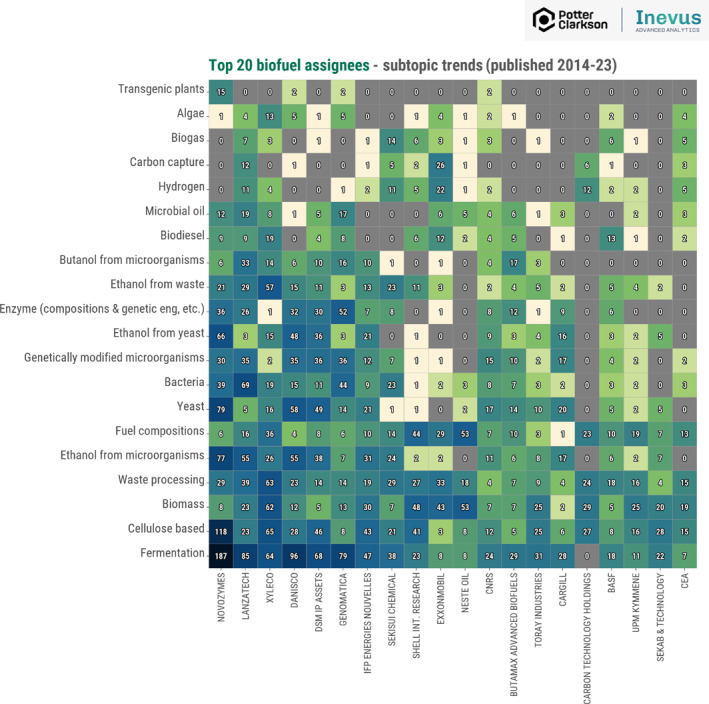
Heatmap showing the distribution of the top 20 biofuel assignees during 2014–2023, split by subtopics as shown in Figure 5.

We can also look at which companies are active in biofuels within each of these subtopics. Figure 6 breaks these subtopics down by assignee (the company or the individual that filed or now owns the patent). Denmark‐based Novozymes has by far the largest number of patent filings in the biofuel space, with a clear focus on fermentation and cellulose. Xyleco is leading the way in algae and ethanol from waste space; Lanzatech and Exxonmobil are active in the hydrogen and carbon capture areas. This kind of analysis can help to highlight potential partners or licensors, those who are active in your area. It can also identify your main competitors, where you may have freedom‐to‐operate issues, for example, or where you might need to design around. Academics can refer to companies operating in the relevant space in grant applications as evidence of likely commercial traction and impact. From an industry perspective, it gives clues as to what really matters most to your main competitors or could identify companies you want to acquire. Equally, it suggests that platform patents around processing technology may outlast first‐generation fuel patents and become relevant for chemical or material applications too.

The full report, which will be available shortly, has similar deep dives on many of the 40 synthetic biology ‘topics’.

## 6 What Shapes Patent Filing Trends?

Even in this short article, and looking at just these few trends, it is clear that patent filing activity in synthetic biology reflects not only advances in science but also the broader context in which that science unfolds. Trends in filings often follow shifts in policy, funding, market confidence and global events rather than research progress alone.

Early policy incentives and subsidy regimes can drive rapid innovation—as seen in the initial biofuels boom—whereas uncertainty or the withdrawal of support can have the opposite effect. External shocks can also have dramatic and lasting impacts: The COVID‐19 pandemic triggered a sharp rise in filings across vaccines, antibodies and diagnostics, much as earlier climate‐driven policies have helped stimulate activity in waste conversion, carbon capture and sustainable materials. These types of events often result in paradigm shifts in how we fundamentally do things, allowing for impact beyond the core area that triggered the need for innovation in the first place (think how the world of vaccines, vaccine delivery, LNPs, mRNA vaccines, origami‐based technology has changed since COVID).

Technological breakthroughs can redefine the innovation landscape almost overnight. The arrival of CRISPR genome editing in 2012, for example, opened entirely new patent categories and research directions, and the crowded patent landscape is now driving innovation in CRISPR alternatives. Likewise, targeted funding programmes—such as the UK's Engineering Biology Mission Hubs—often precede upticks in patenting within newly supported fields, as researchers and companies align with strategic national priorities.

Market conditions also play a key role. In areas dominated by a few large players, or where the economics of scale‐up are challenging, filings may plateau even as research continues apace. The trajectory of biofuels illustrates this well: early optimism and policy backing led to rapid growth, followed by a period of consolidation and slowdown, and now renewed interest as attention turns to waste biomass and hydrogen‐based approaches.

In short, patent trends offer a window into the confidence of the ecosystem—reflecting how policy certainty, technological opportunity and market viability combine to shape where and how innovation happens. Understanding these drivers can help anticipate where future activity in synthetic biology may emerge next.

## 7 Country Insights

**FIGURE 7:**
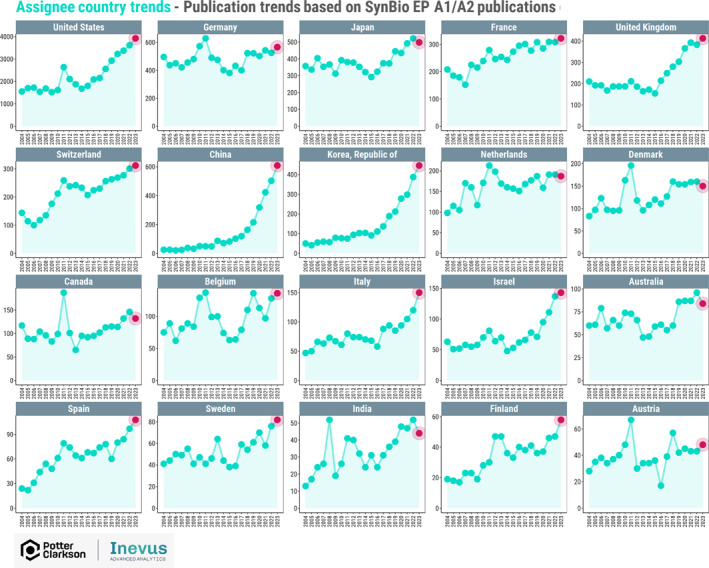
Applicant country of origin of patent publications at the EPO (2004–2023).

By far, most patent applications filed in synthetic biology at the European Patent Office originate from the United States with around 4000 European patent applications publishing in 2023 naming a US‐based applicant. By contrast, the next most prolific country of origin is Germany, with around 550 European patent publications in 2023. Figure 7 sets out the filing trends, based on the country of the applicant. The trend in many countries mirrors that shown for overall synbio patents in Figure 3—with an upward trajectory since 2013. Some countries, such as Spain and Switzerland, although starting from a low base line, have shown a consistent increase in patent filings since 2004 to 2023, with Spain showing a recent steep increases in activity. The United Kingdom looks to be in a very good position and is actively and increasingly filing patent applications in synthetic biology at the EPO. Perhaps somewhat surprisingly, the trend for Germany and Canada is not so positive, being relatively static since 2004, and for Denmark being flat for several years. It will be interesting to see if these trends continue once the report is updated with the most recent 2 years of data.

## 8 University and Private Company Originating Filings

There is much talk, particularly in the United Kingdom, about how much ‘better’ the United States is at spinning out companies and commercialising IP. Accepting that the overall numbers for US originating filings will be much higher than UK originating filings, we were interested in seeing whether the proportion of patents filed by universities (and which may be licenced to a spinout) versus nonuniversity entities was different in the United States to the United Kingdom.

**FIGURE 8:**
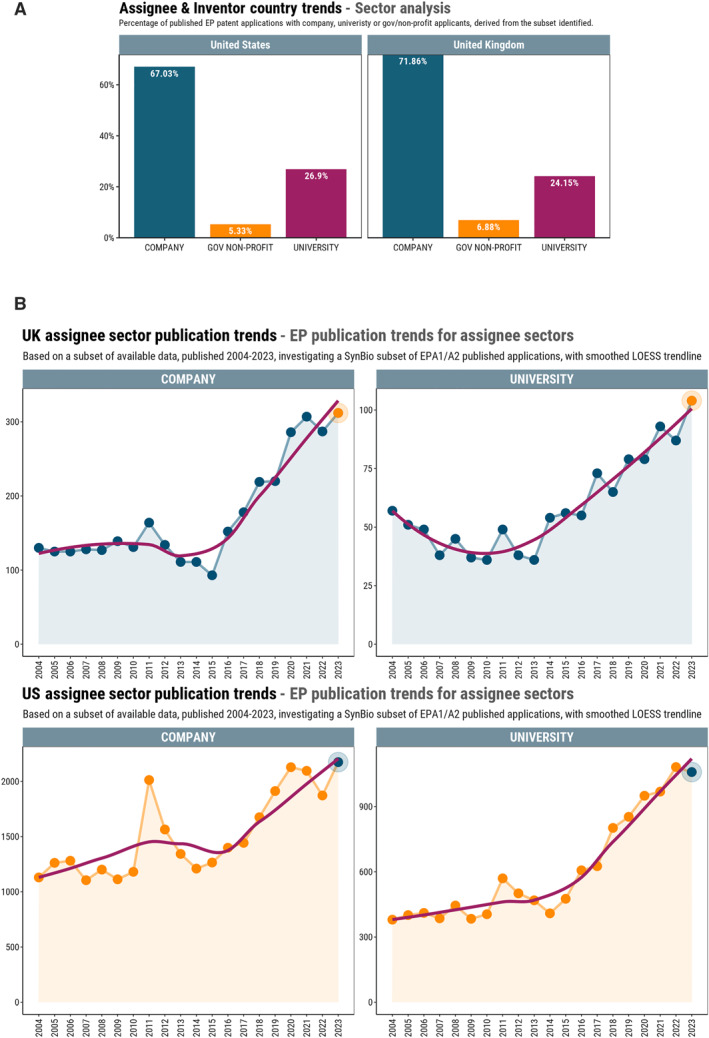
The top 3 types of assignee sectors were investigated for the United Kingdom and, as a comparison country, the United States. Data were retrieved from PATSTAT (the EPO's patent statistical analysis database) for the United States. The sectors were manually assigned for the United Kingdom by processing the data exported from Questel Orbit. It was found that data coverage was not adequate to extend the analysis beyond the United States when relying on PATSTAT data. For example, some assignees had missing data or were ‘UNKNOWN’. The data analysis represents a subset or sample from which approximate conclusions can be drawn as a guide. The analysis applies to assignee country only, inventors are not classified by sectors. (A) Percentage of published European patent applications with a US‐based or UK‐based company, university or gov/nonprofit as the applicant. (B) European patent publication trends where there is a US‐based or UK‐based company or university as the applicant.

Figure 8A shows that in reality, the relative proportion of synbio patents filed by universities, nonprofits and private companies is very similar between the United States and United Kingdom. On the face of it, the synthetic biology innovations are being commercialised to a similar extent by universities and nonuniversity entities across the United States and the United Kingdom. However, a deeper dive into the recent trends shows a different story potentially starting to emerge. In the United States, patent filings in the synbio space from universities are growing faster than from private companies with compound annual growth rates of 11.2% and 6.7%, respectively. Conversely, in the United Kingdom, the values are flipped, with filings from companies growing at a compound annual growth rate of 12.2% versus 7.6% for universities.

It is good for the United Kingdom that both of these values are higher than those for the United States, the main country that the United Kingdom has, to date, compared itself to. It also perhaps tells us something of the different ecosystems of the two countries. Colloquially, the United States is said to be more supportive of spin‐outs, universities file more patents, take more risk and generally have more money than United Kingdom universities. This is probably true, and perhaps it is the combination of these factors, along with a boom in the support offered in the United Kingdom to scientist founders, from various sources, including Wilbe [13] and Carbon 13 [14], that is driving the increase in patent filings (and so innovation) from private companies rather than universities. We will need to dig deeper, but given that the United Kingdom has very few large biotechnology companies that are likely to be filing in the synbio space, the hypothesis is that most of these filings are being made by small start‐ups.

How these trends develop remains to be seen. The relatively recent spin‐out review [15] has encouraged many universities to update their policies to be more founder‐/spin‐out‐friendly so we may expect to see more filings from the universities—provided they are backed up with the cash/staff needed to support the aims of the review.

## 9 Conclusion

Commercialisation of technology arising from synthetic biology relies on many things. It requires a fundamental understanding of molecular biology—how things work—and fundamental research in our academic institutes must continue. It also requires a keen eye and a mind for the ‘what if’ to spot those innovations that do often arise from even the most fundamental piece of research, which in turn requires a basic level of appreciation of inventions and the role that IP plays in getting products out from the lab. It needs a supportive funding environment, including early stage government nonequity funding to the slightly less early stage angel investors and VCs. It needs a streamlined and innovative regulatory system to approve the myriad different products and methods that engineering biology can create. Many of these are being addressed to varying degrees in many different countries. However, it also needs market pull. It needs help to move these amazing synbio products out of small labs onto supermarket shelves into warehouses and building sites. Although the patent analytics presented here are very positive for many applications of synthetic biology, what is currently missing is a systematic mechanism by which markets can either be created for novel synbio products or existing markets be made aware of the advantages of using synthetic biology‐based products in their manufacturing processes or synbio methods to create sustainable versions of incumbent products. Once drivers are established to pull products to market, everyone in the value chain, from founders to investors, will have more confidence, and we will see innovation accelerate.

## Author Contributions

The report was generated by Inevus Advanced Analytics with input from Sara L. Holland and Robert Cogger‐Ward.

## Funding

The author has nothing to report.

## Conflicts of Interest

The author declares no conflicts of interest.

## Data Availability

The data that support the findings of this study are available at https://inevusadvancedanalytics.com/reports/synbio. The raw data are not publicly available due to privacy or ethical restrictions.
